# Verbal Suggestion Modulates the Sense of Ownership and Heat Pain Threshold During the “Injured” Rubber Hand Illusion

**DOI:** 10.3389/fnhum.2022.837496

**Published:** 2022-04-25

**Authors:** Tomoya Tanaka, Kazuki Hayashida, Shu Morioka

**Affiliations:** ^1^Department of Neurorehabilitation, Graduate School of Health Sciences, Kio University, Koryo, Japan; ^2^Department of Rehabilitation, Fukuchiyama City Hospital, Fukuchiyama, Japan; ^3^Neurorehabilitation Research Center, Kio University, Koryo, Japan

**Keywords:** sense of ownership, pain threshold, rubber hand illusion, verbal suggestion, context effect, multisensory integration

## Abstract

The appearance of the self-body influences the feeling that one’s body belongs to oneself, that is, a sense of ownership (SoO) and pain perception. This can be identified by measuring the SoO and pain thresholds after performing the rubber hand illusion (RHI) with an injured rubber hand. The generation of SoO is thought to be caused by multisensory integration of bottom-up factors (vision, proprioceptive, and touch), and by top-down factors, such as the context effect. The appearance is one of the context effects which may become more effective when used simultaneously with other context effects (e.g., verbal suggestion). However, in the RHI, when appearance and other context effects are used simultaneously, the effect is unclear. In this study, we attempted to identify the influence of verbal suggestion on the SoO and heat pain threshold (HPT). As a preliminary step, in Experiment 1, the “normal” rubber hand and “penetrated nail” as injured rubber hand were used to clarify the context effect with appearance alone during RHI (synchronous/asynchronous), which was conducted within-subjects. In Experiment 2, we only used the “penetrated nail” rubber hand to clarify the context effect with verbal suggestion and appearance during RHI. We randomly classified participants into two suggestion groups (“fear” and “no-fear”). The RHI (synchronous/asynchronous) was conducted for each group. In each experiment, the effect of each condition was assessed by subjective measures of SoO, such as questionnaire, and objective measures of SoO, such as proprioceptive drift and electrodermal activity. Following RHI in each condition, HPT was measured. The main finding was that, in the synchronous condition, the “penetrated nail” appearance with “fear” verbal suggestion modulated questionnaire and HPT, but not electrodermal activity. We conclude that the context-included multisensory integration affected the subjective factors because it contains a higher cognitive process by verbal suggestion.

## Introduction

The feeling that one’s body belongs to oneself, that is, the sense of ownership (SoO), is a fundamental aspect of self-consciousness (Gallagher, 2000; Tsakiris, 2010). SoO is the result of the integration of afferent information such as vision, proprioception, and tactile perception and changes flexibly depending on the situation. For example, increasing the SoO of an object with one’s hand (embodiment of a tool) can improve its operability (Cardinali et al., 2021; Odermatt et al., 2021). However, when a person is experiencing mental distress or pain, decreasing SoO can decrease distress perception (Hirakawa et al., 2014; Ataria, 2015; Ten Brink et al., 2021).

The mechanism of SoO has been examined using the rubber hand illusion (RHI) (Botvinick and Cohen, 1998). In the RHI, the participant’s real hand and a rubber hand (i.e., a fake hand made of rubber) are placed in parallel on a table. A partition is positioned between the real and rubber hands, such that only the rubber hand is visible to the participant. When the real and rubber hands are stroked simultaneously, the participant gradually feels as if the rubber hand were a part of their body. This is attributed to the integration of multisensory input (vision, proprioceptive, and touch) through inference depending on the situation (Kilteni et al., 2015; Limanowski, 2021) and is considered the same mechanism as the generation process of the SoO. A similar experimental paradigm has been performed using a virtual arm instead of a rubber hand (virtual hand illusion: VHI) (Slater et al., 2008) and enfacement illusion (Tsakiris, 2008). Moreover, SoO is affected by bottom-up factors (visual, tactile, and proprioception), and also by top-down factors (Synofzik et al., 2008; Tsakiris, 2010). One top-down factor is manipulating the appearance of the rubber hand. For instance, SoO decreases with a swollen (telescopic) and a red-light-illuminated virtual hand (Matamala-Gomez et al., 2020). Furthermore, when a rubber hand with a wound and blood was used in RHI, the pain threshold (Osumi et al., 2014) and pain tolerance (Giummarra et al., 2015) decreased. Similar results were obtained using a virtual hand (Martini et al., 2013; Matamala-Gomez et al., 2020). Thus, top-down factors, such as the appearance of one’s own body, have been shown to affect SoO and pain perception in response to nociceptive stimuli.

Appearance is classified as a “context effect” (Di Blasi et al., 2001). Context effects influence the way the body feels, such as pain (Zou et al., 2016), fatigue (Bottoms et al., 2014), and itch (Bartels et al., 2014). For example, a negative context (e.g., pain, fear) that generates a negative expectation decreases the pain threshold. On the other hand, a positive context (e.g., reassurance) that generates a positive expectation increases the pain threshold. In the context effect, a verbal suggestion is often used because it can change expectations easily and directly (Rossettini et al., 2020). The negative verbal suggestion alone produces a negative context (e.g., pain, fear) and influences body perception, such as decreasing the pain threshold (Colloca et al., 2008; Bajcar et al., 2021). Moreover, context effects are more effective when applying several components collaboratively (Howe et al., 2017; Tinnermann et al., 2017; Olson et al., 2021). Thus, a negative verbal suggestion strongly impacts body perception with a simultaneous negative context appearance (Bublatzky et al., 2019).

Although the context effects using appearance in the RHI and VHI have been widely reported (Tsakiris et al., 2010; Guterstam et al., 2013; Osumi et al., 2014; Martini et al., 2015; Matamala-Gomez et al., 2020), verbal suggestions have rarely been used (Coleshill et al., 2017). For example, context effects like verbal suggestion decreased pain intensity; furthermore, pain intensity was reduced after RHI (Coleshill et al., 2017). However, it is unclear whether verbal suggestion increases pain perception or modulates SoO. In a clinical setting, many patients obtain verbal information from the media and medical professionals (Uritani et al., 2021). The difference in the verbal suggestion content affects pain and psychological outcomes (Louw et al., 2016; Rossettini et al., 2018; Wood and Hendrick, 2019), but the effect on SoO is unclear. Additionally, body illusions such as Virtual reality (VR) have been used as a therapeutic method (Nishigami et al., 2019; Harvie et al., 2020), but there are some problems; SoO toward a virtual body with a positive context appearance does not increase sufficiently, and the degree of SoO differs among individuals (Themelis et al., 2021). In other words, many disorders need to control the degree of illusion to benefit from body illusion. Therefore, if useful verbal suggestions can modulate the degree of illusion, that is, the SoO, it would be meaningful.

The present study aimed to determine the effect of the appearance of a rubber hand with verbal suggestion on SoO and pain threshold in RHI for healthy humans by conducting two experiments. As a preliminary step, in Experiment 1, we examined the effects of the “penetrated nail” appearance (nail) on SoO and heat pain threshold (HPT) relative to the “normal” appearance (normal). Then, in Experiment 2, we examined the effects of the nail rubber hand with verbal suggestions on SoO and HPT, which was the main objective of the experiment. We hypothesized that the nail appearance, which is associated with injury, together with verbal information that causes a fear and pain context, will decrease the SoO and pain threshold during the illusion compared to the control condition, in which verbal suggestions that did not cause fear and pain contexts were used.

## Experiment 1

### Materials and Methods

#### Participants

The inclusion criterion of this study was healthy humans aged between 20 and 39 years. Experiment 1 included 15 healthy participants [6 men, 9 women, 26.8 ± 5.5 years (mean ± SD)]. We calculated the necessary sample size using G*Power: effect size η_*p*_^2^ = 0.51 (Cohen’s *f* = 1.02) (Giummarra et al., 2015), α = 0.05, power = 0.80, number of groups = 2, number of measurements = 2, Corr among rep measures = 0.57 (Osumi et al., 2014), and non-sphericity correction ε = 1 (lower limit). The total required sample size was six participants. However, the sample size of standard RHI experiments ranges from 10–20 to 32 (Lane et al., 2017), so the assumed sample size was too small. Thus, we adopted a sample size of *n* = 15, similar to previous research (Giummarra et al., 2015). We recruited healthy Japanese volunteers aged 20–39 years at Fukuchiyama City Hospital and medical students living in Fukuchiyama City. The exclusion criteria were having a history of psychiatric conditions, neurologic conditions, or chronic pain. Additionally, we excluded participants taking analgesic medication at the time of the experiment. Before the experiment, participants were informed about the experimental procedure, risk of experiments, and compensation. Participants were not informed about the purpose to avoid biased results. Finally, participants provided written consent to participate. The Kio University ethics committee (approval No.: R3-05) and Fukuchiyama City Hospital ethics committee (approval No.: 3-2) approved the study procedures, and the protocol was registered with the UMIN Clinical Trials Registry (UMIN000044233). The experiment was conducted in accordance with the Declaration of Helsinki.

#### Experimental Set-Up

**FIGURE 1:**
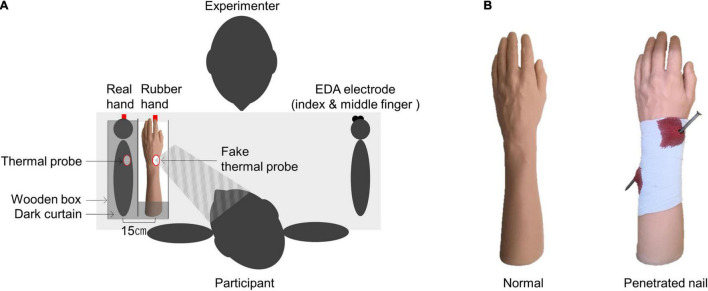
Experimental setting **(A)** and rubber hand **(B)** used in the experiments. We used a normal rubber hand and a nail rubber hand in Experiment 1. In Experiment 2, we used the nail rubber hand only.

The participants were asked to sit in a resting position before a table while wearing clothing that exposed their forearms. A wooden box (length 60.0 cm × width 40.0 cm × height 20.0 cm) was placed on the table, and participants placed their left forearm, palm down, on the outer side of the box. Then, the left index finger was positioned at the red mark in the box (Figure 1A), and the table height was adjusted to a comfortable height for the participant (65.0∼79.0 cm). Inside the same horizontal plane, a rubber hand was placed in parallel. The distance between the index finger of the rubber hand and the real hand was 15 cm, and a dark curtain was placed on the horizontal plane of the box so that participants could not see the real hand. We used a normal rubber hand and nail rubber hand in Experiment 1 (Figure 1B). Based on the previous study finding that the appearance of a shaped object penetrating the body is generally considered to induce pain (Ogino et al., 2007), in this present study, we used the nail rubber hand as the appearance that affects pain perception.

To measure the HPT, a thermal probe was attached to the dorsal side of the left distal forearm (proximal radial styloid process), and a fake thermal probe was attached to the corresponding position of the rubber hand. To measure the electrodermal activity (EDA), two electrodes (Φ2mm) were attached to the palm side of the middle phalanx of the right index and middle fingers. The skin was not pretreated at the attachment site; however, in the case of extremely oily skin, the skin surface was cleaned with alcohol cotton, electrode gel was applied, and electrodes were attached 15 min before the measurements started (Boucsein, 2012). The room temperature was set at approximately 23°C, which is optimal for EDA measurements (Boucsein, 2012). Also, the time of this experiment was between 8:00 and 8:00 p.m. A footswitch was placed at the participant’s right foot to measure the onset time of the illusion (i.e., the time at which participants perceived the rubber hand as their left arm).

The experimenter sat across the table from the participant to apply tactile stimuli to the real and rubber hands using two brushes. We followed the standard RHI procedure (Botvinick and Cohen, 1998; Osumi et al., 2014), with two conditions of tactile stimulation [synchronous stroking condition (synchronous)/asynchronous stroking condition (asynchronous)]. Asynchronous is generally used to block the effects of illusions; that is, it is unlikely that participants feel SoO for the rubber hand. For the tactile stimulation method, we referred to previous studies (Tsakiris and Haggard, 2005; Guterstam et al., 2011). In the synchronous setting, tactile stimuli with synchronized speed and timing were provided to the rubber and real hands. The stimulus rhythm was irregular (500–1000 ms). Conversely, we applied temporally (500–1000 ms) and spatially asynchronous tactile stimuli to the rubber and real hands. Additionally, to reduce the illusion, the positions of the rubber and real hands were slightly mismatched [anatomically, the rubber hand was rotated by 20–30° (Osumi et al., 2014)]. In both conditions, the stimulation site was from the radial-carpal joint on the dorsal side of the left index/middle fingertip.

#### Procedure

Before the experiment, participants reported their age, sex, educational background (years), handedness [the Edinburgh Handedness Inventory (Oldfield, 1971)], and psychological factors [Hospital Anxiety and Depression Scale (HADS) (Zigmond and Snaith, 1983)] (Supplementary Table 1). Moreover, participants practiced the HPT measurement method and the method of using the footswitch. In Experiment 1, a total of four conditions (normal × synchronous, normal × asynchronous, nail × synchronous, nail × asynchronous), including two rubber hands (normal, nail) and two tactile stimuli (synchronous and asynchronous), were conducted on the same participant. That is, we designed a within-subjects experiment using the within-subjects factor × within-subjects factor. The experimental order was counterbalanced. Between each condition, there was a break (<5 min), during which the participants were instructed to move their left upper limb so that the proprioceptive drift and numbness caused by the RHI would not affect the next condition. Before commencing the RHI, participants were instructed to keep both upper limbs still throughout the experiments and to concentrate on the rubber hand.

**FIGURE 2:**
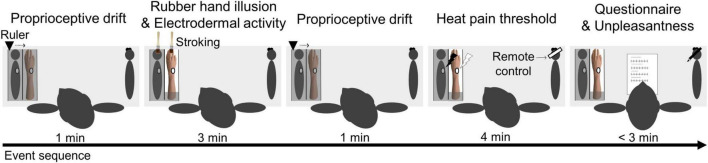
Experiment 1 procedure: (1) proprioceptive drift measurement (pre-RHI); (2) RHI and EDA; (3) proprioceptive drift measurement (post-RHI); (4) HPT measurement; and (5) questionnaire and unpleasantness measurement.

The Experiment 1 sequence was based on a previous study (Osumi et al., 2014). It was designed to include the following steps: (1) proprioceptive drift (pre-RHI) measurement (1 min); (2) RHI and EDA measurement (3 min); (3) proprioceptive drift (post-RHI) measurement (1 min); (4) HPT measurement (4 min); and (5) questionnaire and unpleasantness measurement (< 3 min) (Figure 2).

#### Measurements

In SoO measurements, a questionnaire is used to measure SoO directly, and an indirect objective measure of SoO is generally used (Botvinick and Cohen, 1998; Tsakiris et al., 2010; Guterstam et al., 2011, 2013; Osumi et al., 2014; Giummarra et al., 2015; Lane et al., 2017; Chancel and Ehrsson, 2020). Thus, the questionnaire is a direct subjective measure of SoO. Proprioceptive drift and the electrodermal activity as objective measures of SoO were also used in this study (Chancel and Ehrsson, 2020). Additionally, HPT and the unpleasantness toward rubber hand were measured.

#### Proprioceptive Drift

This is the objective and spatial measure of SoO (Chancel and Ehrsson, 2020). The participant was examined in the position of the participant’s left index finger. Based on a previous study (Osumi et al., 2014), it was conducted in the following order: (1) the top of the box was covered with a dark curtain so that the rubber and real hands were not visible; (2) the experimenter moved the ruler slowly toward the midline from 20 cm to the left of the participant’s left index finger; and (3) when the participant felt that the ruler was located at a certain position of the left index finger of the real hand, the participant verbally informed the experimenter. The experimenter then recorded the position. Notably, the position of the index finger of the real hand was set at 0 cm, and the displacements toward and opposite the rubber hand were defined as “+” and “–,” respectively.

#### Electrodermal Activity Measurement Device and Electrodermal Activity

This is the objective and physiological measure of SoO (Chancel and Ehrsson, 2020). Based on a previous study (Braithwaite et al., 2014), we measured the EDA using EDA measurement equipment (EDR-100; Unique Medical, Tokyo, Japan). This equipment measured the skin resistance level/response of the EDA so that the measured value was smaller and sympathetic nerve activity was higher. AD converters and data recording were performed using a PowerLab and LabChart data acquisition system (PowerLab 800S; ADInstruments, Colorado, United States). The sampling frequency was set to 1000 Hz. After confirming that there was no significant shift in EDA, we commenced the measurement using the 60 s before the tactile stimulation as the baseline and measured EDA throughout the 3 min (Experiment 1) or 5 min (Experiment 2) of the RHI. Moreover, for the EDA analysis, we measured the onset time (the time of commencing tactile stimulation to that when the participant perceived the rubber hand as their own) by stepping on a footswitch placed near the foot (Lane et al., 2017). We then calculated ΔEDA, which is the difference between the mean EDA of 30 s before the onset time and that of 30 s before the start of tactile stimulation (baseline). In Experiment 1, the individual ΔEDAs were standardized as z-scores among the conditions and used for statistical analysis (Eimontaite et al., 2013).

#### Thermal Stimulus Device and Heat Pain Threshold

Heat pain threshold (HPT) was measured using a thermal stimulator (UDH-105; Unique Medical, Tokyo, Japan). Using the method of limits (Osumi et al., 2014), the thermal stimulus started at 32°C, with a 1°C increment per second. Participants were instructed to press the remote control (to stop the temperature increment) by their right hand immediately when they felt pain as a subjective experience. The temperature was recorded as HPT. In each condition, HPT was measured repeatedly four times (every 1 min), and the average value was used for statistical analysis.

#### Questionnaire

This is a subjective measure of SoO (Chancel and Ehrsson, 2020). The questionnaire comprised ownership (SoO of rubber hand), disownership (sense of disowning the real hand), and control (items unaffected by ILLUSION, namely dummy items) (Guterstam et al., 2011; Lane et al., 2017) (Table 1). The questionnaire was translated into Japanese. Each item was rated on a 7-point Likert scale ranging from -3 (*strongly disagree*) to + 3 (*strongly agree*). The mean value in each category was used for statistical analysis.

**TABLE 1:** The questionnaire consists of nine statements divided into three different categories (ownership, disownership, and control).

Statements		Category
Q1	It seemed as though I was feeling the touch in the location where I saw the fake hand being touched.	Ownership
Q2	It seemed as though the fake hand belonged to me.	Ownership
Q3	It seemed as though the fake hand was part of my body.	Ownership
Q4	It seemed as though the touch I felt was caused by the touch on the fake hand.	Ownership
Q5	It appeared (visually) as if the fake hand was drifting toward my own (real) hand.	Control
Q6	It seemed as if I might have more than one left hand or arm.	Control
Q7	I felt as if my real hand no longer belonged to me.	Disownership
Q8	It felt as though my real hand was no longer part of my body.	Disownership
Q9	It felt as though the fake hand replaced my own left hand.	Disownership

#### Unpleasantness Toward the Rubber Hand

We evaluated the unpleasantness toward the rubber hand only in the synchronous condition using a numeric rating scale (NRS): 0 (*no unpleasantness*) to 10 (*worst possible unpleasantness*) (Osumi et al., 2014). This measured how participants perceived the rubber hand.

#### Statistical Analysis

Heat pain threshold (HPT) had a normal distribution (Shapiro–Wilk test: *p* > 0.05), but the sphericity assumption was unmet (Mendoza‘s Multisample Sphericity Test: *p* = 0.028); therefore, we corrected using the Greenhouse-Geisser ε. Next, a 2 (APPEARANCE: normal or nail) × 2 (ILLUSION: synchronous or asynchronous) repeated measures analysis of variance (ANOVA) was used for HPT.

In the SoO outcomes, because the questionnaire (each sub-item), proprioceptive drift, and onset time did not have a normal distribution (Shapiro–Wilk test: *p* < 0.05), a 2 (APPEARANCE: normal or nail) × 2 (ILLUSION: synchronous or asynchronous) repeated measures ANOVA was used with aligned rank transform (ART) (Wobbrock et al., 2011). Moreover, the EDA had a normal distribution (Shapiro–Wilk test: *p* > 0.05), and the sphericity assumption was met (Mendoza’s Multisample Sphericity Test: *p* > 0.05); therefore, a 2 (APPEARANCE: normal or nail) × 2 (ILLUSION: synchronous or asynchronous) repeated measures ANOVA was used. Notably, participants (*n* = 5) had an onset time of <30 s; thus, EDA analysis was performed with *n* = 10, excluding these.

Because unpleasantness did not have a normal distribution (Shapiro–Wilk test: *p* < 0.05), the Wilcoxon signed-rank test was used. Additionally, the association between HPT and questionnaire ownership was analyzed using Spearman’s rank correlation.

To help with the interaction of the results from this study, effect sizes were calculated based on Cliff’s δ [< 0.147 = negligible, < 0.33 = small, < 0.474 = medium, otherwise = large (Romano et al., 2006)]. The significance level was set at *p* < 0.05, and R (ver.4.0.0) was used for statistical analysis.

### Results

#### Heat Pain Threshold

**FIGURE 3:**
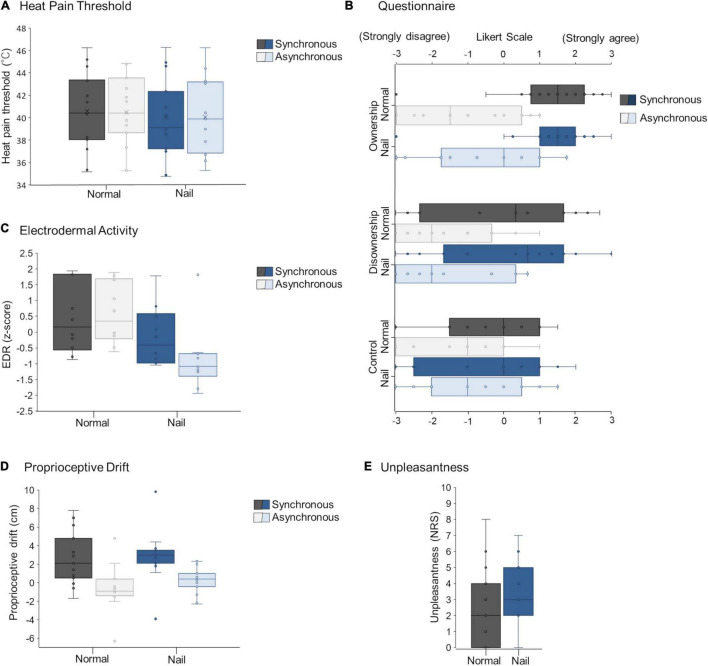
HPT **(A)**, questionnaire **(B)**, EDA **(C)**, proprioceptive drift **(D)**, and unpleasantness **(E)** in Experiment 1.

ANOVA results showed no interaction between APPEARANCE and ILLUSION [*F*_*1,14*_ = 0.10, *p* = 0.76, η_*p*_^2^ = 0.0070; normal × syncronous, 38.76–42.41°C (95% confidence interval); normal × asyncronous, 38.66°C–42.32°C; nail × syncronous, 38.17°C–41.82 °C; nail × asyncronous, 38.21°C–41.87 °C]. There was also no main effect of APPEARANCE (normal vs. nail: *F*_*1,14*_ = 1.33, *p* = 0.27, η_*p*_^2^ = 0.087) or ILLUSION (synchronous vs. asynchronous: *F*_*1,14*_ = 0.02, *p* = 0.90, η_*p*_^2^ = 0.0012) (Figure 3A).

#### Questionnaire

Concerning ownership, ANOVA using ART showed no interaction between ILLUSION and APPEARANCE (*F*_*1,42*_ = 1.26, *p* = 0.27, η_*p*_^2^ = 0.029). There was also a significant main effect of ILLUSION (*F*_*1,42*_ = 42.78, *p* < 0.001, η_*p*_^2^ = 0.51), which was higher during synchronous tactile stimuli than during asynchronous tactile stimuli, but not main effect of APPEARANCE (*F*_*1,42*_ = 1.25, *p* = 0.29, η_*p*_^2^ = 0.027) (Figure 3B).

Regarding disownership, ANOVA using ART showed no interaction between ILLUSION and APPEARANCE (*F*_*1,42*_ = 0.016, *p* = 0.90, η_*p*_^2^ < 0.001). There was also a significant main effect of ILLUSION (*F*_*1,42*_ = 33.42, *p* < 0.001, η_*p*_^2^ = 0.44), which was higher during synchronous tactile stimuli than during asynchronous tactile stimuli, but not the main effect of APPEARANCE (*F*_*1,42*_ = 0.10, *p* = 0.75, η_*p*_^2^ = 0.0024) (Figure 3B).

Concerning the control, ANOVA using ART showed no interaction between ILLUSION and APPEARANCE (*F*_*1,42*_ = 0.61, *p* = 0.44, η_*p*_^2^ = 0.014). In addition, there was a significant main effect of ILLUSION (*F*_*1,42*_ = 9.21, *p* = 0.0041, η_*p*_^2^ = 0.18; see Limitations in Discussion), which was higher during synchronous tactile stimuli than during asynchronous tactile stimuli, but not the main effect of APPEARANCE (*F*_*1,42*_ = 1.64, *p* = 0.21, η_*p*_^2^ = 0.038) (Figure 3B).

#### Electrodermal Activity

Regarding EDA, there was no interaction between ILLUSION and APPEARANCE (*F*_*1,27*_ = 1.72, *p* = 0.20, η_*p*_^2^ = 0.060). There was a significant main effect of APPEARANCE (*F*_*1,27*_ = 10.33, *p* = 0.0034, η_*p*_^2^ = 0.28) that was lower (i.e., increasing activity of sympathetic nerve) on the nail rubber hand than the normal rubber hand, but there was no main effect of ILLUSION (*F*_*1,27*_ = 0.73, *p* = 0.40, η_*p*_^2^ = 0.026) (Figure 3C).

Notably, the onset time was used for this EDA analysis, and there was a significant main effect of ILLUSION (*p* < 0.001). There was no main effect for APPEARANCE and no interaction between ILLUSION and APPEARANCE.

#### Proprioceptive Drift

There was no interaction between ILLUSION and APPEARANCE (*F*_*1,42*_ = 0.35, *p* = 0.56, η_*p*_^2^ = 0.0082) (Figure 3D). There was a significant main effect of ILLUSION (*F*_*1,42*_ = 60.21, *p* < 0.001, η_*p*_^2^ = 0.59), which was longer during synchronous tactile stimuli than asynchronous tactile stimuli and APPEARANCE (*F*_*1,42*_ = 4.34, *p* = 0.043, η_*p*_^2^ = 0.094), which was as longer on the nail rubber hand than the normal rubber hand (Figure 3D).

#### Unpleasantness

There was no significant difference between the normal and nail conditions [*V* = 54, *p* = 0.071, Cliff’s δ = 0.34; normal, 2.3 ± 0.64 (mean ± SE); nail, 3.4 ± 0.48] (Figure 3E).

#### Relationship Between Heat Pain Threshold and Ownership of Questionnaire

**FIGURE 4:**
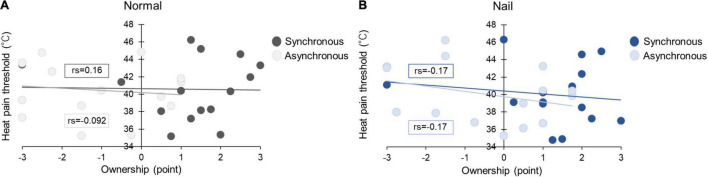
Relationship between ownership of questionnaire and HPT in Experiment 1 **(A,B)**.

The data showed no significant correlation between HPT and ownership in normal × synchronous (rs = 0.16, *p* = 0.57), normal × asynchronous (rs = –0.092, *p* = 0.74), nail × synchronous (rs = –0.17, *p* = 0.55), and nail × asynchronous (rs = –0.17, *p* = 0.55) (Figure 4).

### Brief Discussion

In Experiment 1, we examined the conditions of SoO and HPT when only the nail rubber hand was used relative to the normal rubber hand. The results showed that the questionnaire and HPT for the nail rubber hand might be no different than those for the normal rubber hand. Furthermore, the EDA increased with the appearance of the nail rubber hand regardless of the RHI. This result may reflect the emotional response to nail appearance.

In the results of proprioceptive drift, there are two possible reasons for the larger effect of the nail rubber hand relative to the normal rubber hand. The first is the effect of the emotional response described above. The emotional response during the RHI increases the proprioceptive drift (Engelen et al., 2017). The second is the effect of spatial congruence between blood on the nail rubber hand and the thermal probe on the real hand. People associate color and temperature with each other, such as red with warmth or blue with cold (Bennett and Rey, 1972). In the RHI and VHI, red light on a rubber hand and warmth by thermal prove on the real hand promote the illusion (Trojan et al., 2018; Cordier et al., 2020). In this study, we believe that the spatial consistency of the blood and thermal probes increased the proprioceptive drift, which is a spatial factor.

Next, in Experiment 2, we examined the effect of adding verbal information to the nail rubber hand, which has no change in subjective factors, such as questionnaire and HPT.

## Experiment 2

### Materials and Methods

#### Participants

Experiment 2 included 30 healthy participants different from those in Experiment 1. Because there was no proper previous study, the effect size was estimated to be moderate (Cohen, 1992). The sample size was calculated using G*Power: effect size *f* = 0.25 as medium effect size (Cohen, 1992), α = 0.05, power = 0.80, number of groups = 2, number of measurements = 2, Corr among rep measures = 0.57, Nosphericity correction ε = 1. A total of 30 participants were required. The recruitment of participants, inclusion and exclusion criteria, explanation before the experiment, and ethical statements are identical to Experiment 1.

#### Experimental Set-Up

We employed an identical setup to Experiment 1. However, in Experiment 2, we used only the nail rubber hand and excluded the normal rubber hand.

#### Procedure

In Experiment 2, we modified four points of the experimental procedure from Experiment 1. All other procedures were identical to Experiment 1.

First, we designed a within-between-subjects experiment. As a between-subjects factor, participants were randomly allocated one of two verbal suggestions: (1) fear verbal suggestion condition [fear group *n* = 15: 5 men, 10 women, 27.5 ± 4.4 years (mean ± SD)]; (2) no-fear verbal suggestion (no-fear group *n* = 15: 6 men, 9 women, 26.0 ± 4.5 years). As a within-subjects factor, two types of tactile stimuli (synchronous and asynchronous) were used. Notably, there was no significant difference between the no-fear and fear groups regarding age, sex, educational background, handedness, and psychological factors (HADS: total, anxiety, depression) (*p* > 0.05) (Supplementary Table 1).

**FIGURE 5:**
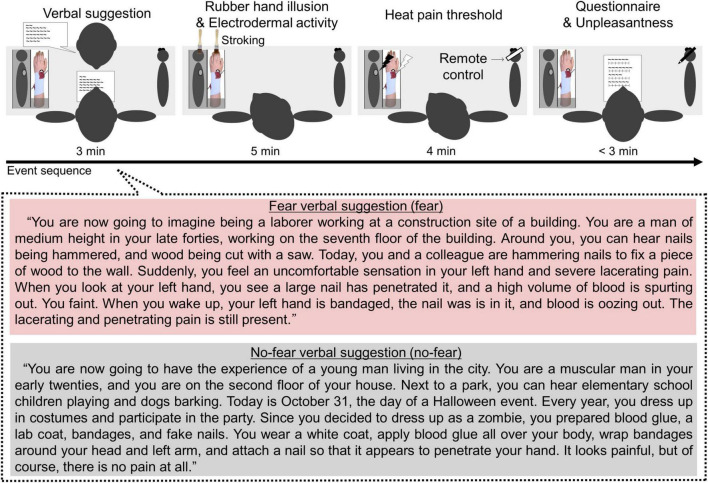
Experiment 2 procedure: (1) verbal suggestion; (2) RHI and EDA measurement; (3) HPT measurement; (4) questionnaire and unpleasantness measurement [(B) Top]. Verbal suggestion: fear verbal suggestion (fear) and no-fear verbal suggestion (no-fear) comprised approximately 280 Japanese characters each [(B) Bottom].

Second, before the RHI, participants were provided verbal information (i.e., verbal suggestion) that either caused or did not cause a fear context (Figure 5). A fear verbal suggestion (fear) and no fear verbal suggestion (no fear) were used to create or avoid feelings of fear toward the nail rubber hand, respectively. Verbal suggestions that described a nail rubber hand scenario using everyday words were created by the author (TT) and consisted of approximately 280 Japanese characters each. Before use in the experiment, we conducted a pilot survey to determine whether the suggestions caused feelings of fear in another 20 healthy participants [12 men, 8 women, 27.8 ± 4.2 years (mean ± SD)]. When reading and imagining the verbal suggestions on the paper, they answered the degree of fear using an NRS: 0 (*no fear*) to 10 (*worst fear imaginable*). The result showed that the fear verbal suggestion was significantly more fearful [Wilcoxon signed-rank test: *p* < 0.001, fear, 7.6 ± 0.4 (mean ± SE) vs. no-fear, 1.1 ± 0.5]. Initially, the verbal suggestion was read aloud by the experimenter. To facilitate understanding, participants were also presented with written text. After being presented with either verbal suggestion (fear or no-fear), participants were presented with a nail rubber hand and informed that “This (the rubber hand) is a reproduction of the left arm” Therefore, we initiated the RHI.

Third, we were interested in subjective measures of SoO (i.e., questionnaires) and HPT; thus, proprioceptive drift was not conducted in Experiment 2. This is because we contend that measuring the proprioceptive drift would reduce the degree of illusion (Perepelkina et al., 2018; Pfister et al., 2021) and might affect the evaluation of the HPT.

Fourth, the RHI was extended from 3 to 5 min. RHI reportedly increases with time (Perepelkina et al., 2018; Pfister et al., 2021). Thus, to measure the HPT under the condition of heightened illusion, we extended the duration of the RHI.

Therefore, the experimental sequence was designed to include the following steps: (1) verbal suggestion (3 min), (2) RHI and EDA measurement (5 min), (3) HPT measurement (4 min), and (5) questionnaire and unpleasantness measurement (< 3 min) (Figure 5).

#### Measurement

The measurements were based on Experiment 1. However, regarding the data analysis method, in Experiment 2, we used raw ΔEDA for statistical analysis, but not z-scores. This is because the same participant was measured twice, and standardization by z-score between conditions was not possible.

#### Statistical Analysis

The HPT was normally distributed (Shapiro–Wilk test: *p* > 0.05) and met the sphericity assumption (Mendoza’s Multisample Sphericity Test: *p* = 0.69). Thus, a 2 (VERBAL: no-fear or fear) × 2 (ILLUSION: synchronous or asynchronous) between-subjects factor × within-subjects factor two-way ANOVA was used.

The questionnaire (each sub-item) and EDA (and onset time) did not have a normal distribution (Shapiro–Wilk test: *p* < 0.05); therefore, we used 2 (VERBAL: no-fear or fear) × 2 (ILLUSION: synchronous or asynchronous) ANOVA using ART. When an interaction was calculated, the Mann–Whitney U test (Bonferroni adjusted) was performed as a *post hoc* test. Notably, participants (fear group, *n* = 4; no-fear group, *n* = 3) had an onset time of < 30 s; thus, EDA analysis was performed with *n* = 12 (no-fear group) and *n* = 11 (fear group), excluding these.

Because unpleasantness did not have a normal distribution (Shapiro–Wilk test: *p* < 0.05), the Mann–Whitney U test was used. In addition, the association between HPT and questionnaire ownership was analyzed using Spearman’s rank correlation.

To clarify the interaction of the results from this study, effect sizes were calculated based on Cliff’s δ [< 0.147 = negligible, < 0.33 = small, < 0.474 = medium, otherwise = large (Romano et al., 2006)]. The significance level was set at *p* < 0.05, and R (ver.4.0.0) was used for statistical analysis.

### Results

#### Heat Pain Threshold

**FIGURE 6:**
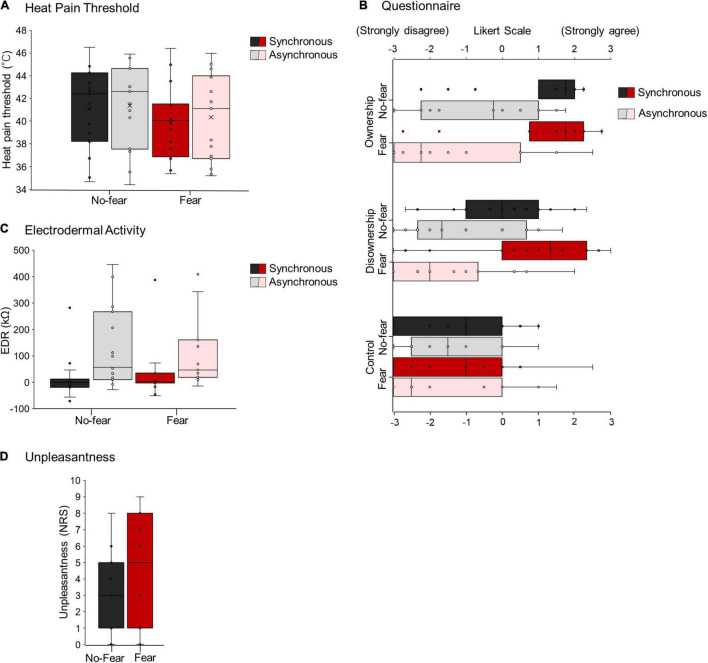
HPT **(A)**, questionnaire **(B)**, EDA **(C)**, and unpleasantness **(D)** in Experiment 2.

ANOVA results showed no interaction between VERBAL and ILLUSION [*F*_*1,28*_ = 0.17, *p* = 0.69, η_*p*_^2^ = 0.0059; no-fear × syncronous, 39.15–42.99°C (95% confidence interval); no-fear × asyncronous, 39.43–43.27°C; fear × syncronous, 37.93–41.77°C; fear × asyncronous, 38.41–42.25°C]. There was also no main effect of VERBAL (fear vs. no-fear: *F*_*1,28*_ = 0.74, *p* = 0.40, η_*p*_^2^ = 0.026) or ILLUSION (synchronous vs. asynchronous: *F*_*1,28*_ = 2.32, *p* = 0.14, η_*p*_^2^ = 0.076) (Figure 6A).

#### Questionnaire

Regarding ownership, two-way ANOVA using ART showed no interaction between ILLUSION and VERBAL (*F*_*1,28*_ = 2.56, *p* = 0.12, η_*p*_^2^ = 0.084). There was also a significant main effect of ILLUSION (*F*_*1,28*_ = 48.40, *p* < 0.001, η_*p*_^2^ = 0.63), which was higher during synchronous tactile stimuli than during asynchronous tactile stimuli, but not the main effect of VERBAL (*F*_*1,28*_ = 0.78, *p* = 0.39, η_*p*_^2^ = 0.027) (Figure 6B).

Regarding disownership, two-way ANOVA using ART showed a significant interaction between ILLUSION and VERBAL (*F*_*1,28*_ = 4.86, *p* = 0.036, η_*p*_^2^ = 0.15). The *post hoc* test revealed a significant difference between synchronous and asynchronous conditions under fear (*p* = 0.017), which was higher during synchronous than asynchronous conditions. There was also a significant main effect of ILLUSION (*F*_*1,28*_ = 22.42, *p* < 0.001, η_*p*_^2^ = 0.45), which was higher during synchronous tactile stimuli than during asynchronous tactile stimuli, but not the main effect of VERBAL (*F*_*1,28*_ = 0.018, *p* = 0.29, η_*p*_^2^ < 0.001) (Figure 6B).

Concerning the control, two-way ANOVA using ART showed no interaction between ILLUSION and VERBAL (*F*_*1,28*_ = 0.91, *p* = 0.35, η_*p*_^2^ = 0.032). In addition, there was no main effect of ILLUSION (*F*_*1,28*_ = 2.01, *p* = 0.17, η_*p*_^2^ = 0.067) or VERBAL (*F*_*1,28*_ = 0.20, *p* = 0.66, η_*p*_^2^ = 0.0072) (Figure 6B).

#### Electrodermal Activity

There was no interaction between ILLUSION and VERBAL (*F*_*1,21*_ = 0.31, *p* = 0.59, η_*p*_^2^ = 0.014). There was a significant main effect of ILLUSION (*F*_*1,21*_ = 9.55, *p* = 0.0056, η_*p*_^2^ = 0.24), which was lower (i.e., increasing activity of sympathetic nerve) on synchronous tactile stimuli than asynchronous tactile stimuli, but not the main effect of VERBAL (*F*_*1,21*_ = 0.16, *p* = 0.69, η_*p*_^2^ = 0.0078) (Figure 6C).

Notably, the onset time was used for this EDA analysis, and there was a significant main effect of ILLUSION (*p* < 0.001). There was no main effect for VERBAL and no interaction between ILLUSION and VERBAL in Experiment 2.

#### Unpleasantness

There was no significant difference between no-fear and fear [U = 83.5, p-value = 0.2338, Cliff’s δ = 0.26; no-fear, 3.2 ± 0.60 (mean ± SE); fear, 4.5 ± 0.87] (Figure 6D).

#### Relationship Between Heat Pain Threshold and Ownership of Questionnaire

**FIGURE 7:**
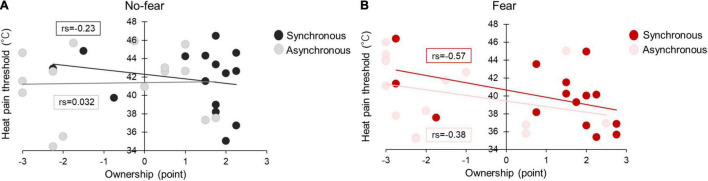
Relationship between ownership of questionnaire and HPT in Experiment 2 **(A,B)**.

The data showed a significant negative correlation between HPT and ownership in fear × synchronous (rs = –0.57, *p* = 0.025), but not for fear × asynchronous (rs = –0.38, *p* = 0.17), no-fear × synchronous (rs = –0.23, *p* = 0.41), and no-fear × asynchronous (rs = 0.032, *p* = 0.91) (Figure 7).

## Discussion

This study aimed to determine the effect of rubber hand appearance with verbal suggestion on SoO and pain threshold in RHI. We hypothesized that the nail appearance with the fear verbal suggestion during the synchronous condition would reduce SoO and HPT relative to the no-fear verbal suggestion. Contrary to the hypothesis, the presentation of fear verbal suggestions did not decrease SoO and HPT during the synchronous condition. Conversely, fear verbal suggestions during the synchronous condition produced higher disownership in the questionnaire. Moreover, correlation analysis revealed, that when presenting the fear verbal suggestion during the synchronous condition, the higher the SoO toward the nail rubber hand, the lower the HPT. These correlations and higher disownership may show that context effect by fear verbal suggestion as a higher cognitive process modulates multisensory integration; “context-included multisensory integration (CIMI)” by verbal suggestion may affect the subjective measures such as questionnaire and HPT.

In the RHI, visual information (see rubber hand position) suppresses proprioceptive information (perceive real hand position), causing the SoO of the rubber hand to increase, whereas the SoO of the real hand decreases (Limanowski, 2021). Thus, ownership and disownership are two sides of the same coin in the questionnaire. In this study, the inconsistency between ownership and disownership, that is, the fear verbal suggestion with the nail rubber hand increased disownership, but not ownership, during the synchronous condition. This may be affected by the measurement method. Regarding the RHI, the SoO toward the rubber hand first increases after the onset of RHI, and that toward the real hand decreases with delay (Lane et al., 2017). Accordingly, disownership was lower than ownership when scored by the Likert scale (Lane et al., 2017). In Experiment 1, ownership had already increased to some extent by nail appearance alone in the synchronous condition. Thus, in Experiment 2, the context effect by fear verbal suggestion was not reflected in ownership. However, in Experiment 1, disownership was not sufficiently increased by nail appearance alone during the synchronous condition. Thus, in Experiment 2, the context effect by fear verbal suggestions was reflected in disownership. As described, although the context effect by fear verbal suggestion was not reflected in ownership by this measuring method, it was shown to affect a part of the subjective measures of SoO, supporting the concept that SoO is affected by the top-down factor (Synofzik et al., 2008; Tsakiris, 2010).

The effect of the top-down factor on SoO in body illusion has been well reported as a correlation between subjective measures (Nierula et al., 2017; Matamala-Gomez et al., 2019, 2020; Ma et al., 2021). In Experiments 1 and 2, a significant correlation between HPT and ownership of the questionnaire was found only when presenting the fear verbal suggestion during the synchronous condition. When the top-down factors are presented, there was a strong correlation between the subjective measure in the VHI (Ma et al., 2021). Additionally, in body illusion using negative context appearance (Nierula et al., 2017; Matamala-Gomez et al., 2019, 2020), the questionnaire and HPT were negatively correlated. In this study, combining the fact that various personal factors (e.g., personality) influence the susceptibility to context effect (Corsi and Colloca, 2017; Kern et al., 2020), it can be interpreted that the fear context by a fear verbal suggestion influenced subjective measures in body illusion and the degree of influence was individually different. The participants who were more sensitive to the fear context by a fear verbal suggestion showed a higher SoO toward the nail rubber hand and had lower HPT. Alternatively, the less sensitive participants showed lower SoO and higher HPT. In summary, there may be individual differences in CIMI by verbal suggestion, and the effect may appear in the subjective measures.

However, no effect of verbal suggestion was found in the EDA results. The inconsistency between the subjective measures and objective measures was reported in body illusion studies (Riemer et al., 2015; Chancel and Ehrsson, 2020; Ma et al., 2021). Additionally, the context has been reported to affect only subjective measures, not objective ones (Benedetti et al., 2003; Peerdeman et al., 2015). Thus, the effect of adding context by verbal suggestion, which is a higher cognitive process during RHI, that is, CIMI may be more likely to affect subjective measures of SoO.

Notably, the disownership of the questionnaire and correlation results showed that the fear verbal suggestion, not a no-fear verbal suggestion, modulates the subjective measures of SoO. In other words, only the fear context caused by verbal suggestion modulated multisensory integration. This is because both fear verbal suggestion and nail appearance have the same elements that produce negative contexts, such as fear and pain. In the context effect, previous studies reported that the effect becomes stronger when the same components are combined (Sessa et al., 2014; Meconi et al., 2018; Bublatzky et al., 2019, 2020). Thus, we believe that the contextual congruence between appearance and verbal suggestion may promote self-consciousness-related multisensory integration.

Despite the significant findings, this study had some limitations. First, notably, the within-between-subjects design was adopted in Experiment 2, and Experiment 1 and Experiment 2 used different participants. Thus, the effects of individual differences in pain threshold and SoO may be related to the results obtained in this study. Second, unlike the previous study (Osumi et al., 2014; Giummarra et al., 2015), in Experiment 1, the nail appearance affected only objective factors, not subjective factors. Therefore, if it is true that context effects have a higher cognitive process influence on subjective measures, the nail appearance may have had a stronger effect by the bottom-up factors, such as vision and proprioception, than the context effect as a top-down factor. Third, in Experiment 1, there was a significant main effect of ILLUSION on the control in the questionnaire. However, control items tended to have smaller differences between synchronous and asynchronous conditions relative to other items (i.e., ownership and disownership) in Experiment 1. Additionally, the effect size was smaller than the other items. In a previous study, there was a significant main effect of ILLUSION in several control items (Fiorio et al., 2011). Additionally, the median value of control items was approximately one point higher in the synchronous condition than in the asynchronous condition (Kalckert et al., 2019). This was consistent with our results and did not cause major problems in interpreting the results.

## Clinical Applications

In clinical studies, pain (Hirakawa et al., 2014; Ten Brink et al., 2021) and mental disorders (Ataria, 2015) have been reported to have lower SoO. Furthermore, persistently decreasing SoO may interfere with recovery. Based on this present result, verbal suggestions (e.g., patient education, patient–practitioner communication) to such disorders may be able to increase their SoO. However, it remains unclear whether a fear context-specific verbal suggestion or verbal suggestion that generates the same context as body appearance is responsible for this effect.

In recent years, the rehabilitation of pain disorders using VR has been produced. Moreover, the use of VR that depicts a positive context for the body, such as muscular appearance (Nishigami et al., 2019), boxers (Harvie et al., 2020), and American comic characters (Harvie et al., 2020), can reduce pain. However, SoO is not sufficiently induced in the body in such VR (Themelis et al., 2021). Thus, adding verbal information to explain the situation and creating a narrative may facilitate multisensory integration and improve SoO.

## Conclusion

The context effect using fear verbal suggestion modulated subjective measure of SoO and HPT during RHI with the nail rubber hand. Because it is the multisensory integration included context as a higher cognitive process, it may affect the subjective factors, not objective ones. These findings provide fundamental insight into the fact that verbal information, which is used casually in clinical settings, can also affect the way the body feels. Additionally, it may apply to rehabilitation using VR, and further research should be conducted in the future.

## Data Availability Statement

The raw data supporting the conclusions of this article will be made available by the authors, without undue reservation.

## Ethics Statement

The studies involving human participants were reviewed and approved by the Kio University Ethics Committee (R3-05) and the Fukuchiyama City Hospital Ethics Committee (3-2). The patients/participants provided their written informed consent to participate in this study.

## Author Contributions

TT conceived and designed the study, acquired, analyzed, and interpreted the data. KH critically revised the manuscript for important intellectual content. SM supervised the study. All authors contributed to the article and approved the submitted version.

## Conflict of Interest

The authors declare that the research was conducted in the absence of any commercial or financial relationships that could be construed as a potential conflict of interest.

## Publisher’s Note

All claims expressed in this article are solely those of the authors and do not necessarily represent those of their affiliated organizations, or those of the publisher, the editors and the reviewers. Any product that may be evaluated in this article, or claim that may be made by its manufacturer, is not guaranteed or endorsed by the publisher.

## References

[B1] Ataria Y. (2015). Sense of ownership and sense of agency during trauma. Phenomenol. *Cogn. Sci.* 14 199–212. 10.1007/s11097-013-9334-y

[B2] Bajcar E. A. Wiercioch-Kuzianik K. Farley D. Buglewicz E. Paulewicz B. Bąbel P. (2021). Order does matter: the combined effects of classical conditioning and verbal suggestions on placebo hypoalgesia and nocebo hyperalgesia. *Pain* 162:22372245. 10.1097/j.pain.0000000000002211 34256381 PMC8280968

[B3] Bartels D. J. van Laarhoven A. I. Haverkamp E. A. Wilder-Smith O. H. Donders A. R. van Middendorp H. (2014). Role of conditioning and verbal suggestion in placebo and nocebo effects on itch. *PLoS One* 9:e91727. 10.1371/journal.pone.0091727 24646924 PMC3960153

[B4] Benedetti F. Pollo A. Lopiano L. Lanotte M. Vighetti S. Rainero I. (2003). Conscious expectation and unconscious conditioning in analgesic, motor, and hormonal placebo/nocebo responses. *J. Neurosci.* 23 4315–4323. 10.1523/JNEUROSCI.23-10-04315.2003 12764120 PMC6741114

[B5] Bennett C. A. Rey P. (1972). What’s so hot about red? *Hum. Factors* 14 149–154. 10.1177/001872087201400204 5022474

[B6] Bottoms L. Buscombe R. Nicholettos A. (2014). The placebo and nocebo effects on peak minute power during incremental arm crank ergometry. *Eur. J. Sport Sci.* 14 362–367. 10.1080/17461391.2013.822564 23889363

[B7] Botvinick M. Cohen J. (1998). Rubber hands ‘feel’ touch that eyes see. *Nature* 391:756. 10.1038/35784 9486643

[B8] Boucsein W. (2012). *Electrodermal Activity*, 2nd Edn. Heidelberg: Springer Science & Business Media, 10.1007/978-1-4614-1126-0

[B9] Braithwaite J. J. Broglia E. Watson D. G. (2014). Autonomic emotional responses to the induction of the rubber-hand illusion in those that report anomalous bodily experiences: evidence for specific psychophysiological components associated with illusory body representations. *J. Exp. Psychol. Hum. Percept. Perform.* 40 1131–1145. 10.1037/a0036077 24635201

[B10] Bublatzky F. Kavcıoğlu F. Guerra P. Doll S. Junghöfer M. (2020). Contextual information resolves uncertainty about ambiguous facial emotions: Behavioral and magnetoencephalographic correlates. *Neuroimage* 215:116814. 10.1016/j.neuroimage.2020.116814 32276073

[B11] Bublatzky F. Riemer M. Guerra P. (2019). Reversing threat to safety: incongruence of facial emotions and instructed threat modulates conscious perception but not physiological responding. *Front. Psychol.* 10:2091. 10.3389/fpsyg.2019.02091 31572272 PMC6753879

[B12] Cardinali L. Zanini A. Yanofsky R. Roy A. C. de Vignemont F. Culham J. C. (2021). The toolish hand illusion: embodiment of a tool based on similarity with the hand. *Sci. Rep.* 11:2024. 10.1038/s41598-021-81706-6 33479395 PMC7820319

[B13] Chancel M. Ehrsson H. H. (2020). Which hand is mine? discriminating body ownership perception in a two-alternative forced-choice task. *Atten. Percept. Psychophys.* 82 4058–4083. 10.3758/s13414-020-02107-x 32856222 PMC7593318

[B14] Cohen J. (1992). A power primer. *Psychol. Bull.* 112 155–159. 10.1037//0033-2909.112.1.155 19565683

[B15] Coleshill M. J. George D. N. Mazzoni G. (2017). Placebo analgesia from a rubber hand. *J. Pain* 18 1067–1077. 10.1016/j.jpain.2017.04.004 28455248

[B16] Colloca L. Sigaudo M. Benedetti F. (2008). The role of learning in nocebo and placebo effects. *Pain* 136 211–218. 10.1016/j.pain.2008.02.006 18372113

[B17] Cordier L. Fuchs X. Herpertz S. Trojan J. Diers M. (2020). Synchronous stimulation with light and heat induces body ownership and reduces pain perception. *J. Pain* 21 700–707. 10.1016/j.jpain.2019.10.009 31698132

[B18] Corsi N. Colloca L. (2017). Placebo and Nocebo Effects: The advantage of measuring expectations and psychological factors. *Front. Psychol.* 8:308. 10.3389/fpsyg.2017.00308 28321201 PMC5337503

[B19] Di Blasi Z. Harkness E. Ernst E. Georgiou A. Kleijnen J. (2001). Influence of context effects on health outcomes: a systematic review. *Lancet* 357 757–762. 10.1016/s0140-6736(00)04169-6 11253970

[B20] Eimontaite I. Nicolle A. Schindler I. Goel V. (2013). The effect of partner-directed emotion in social exchange decision-making. *Front. Psychol.* 4:469. 10.3389/fpsyg.2013.00469 23898313 PMC3722477

[B21] Engelen T. Watson R. Pavani F. de Gelder B. (2017). Affective vocalizations influence body ownership as measured in the rubber hand illusion. *PLoS One* 12:e0186009. 10.1371/journal.pone.0186009 28982176 PMC5628997

[B22] Fiorio M. Weise D. Önal-Hartmann C. Zeller D. Tinazzi M. Classen J. (2011). Impairment of the rubber hand illusion in focal hand dystonia. *Brain* 134(Pt 5) 1428–1437. 10.1093/brain/awr026 21378099

[B23] Gallagher S. (2000). Philosophical conceptions of the self: implications for cognitive science. *Trends Cogn. Sci.* 4 14–21. 10.1016/s1364-6613(99)01417-5 10637618

[B24] Giummarra M. J. Georgiou-Karistianis N. Verdejo-Garcia A. Gibson S. J. (2015). Feeling the burn: when it looks like it hurts, and belongs to me, it really does hurt more. *Conscious. Cogn.* 36 314–326. 10.1016/j.concog.2015.07.010 26232354

[B25] Guterstam A. Gentile G. Ehrsson H. H. (2013). The invisible hand illusion: multisensory integration leads to the embodiment of a discrete volume of empty space. *J. Cogn. Neurosci.* 25 1078–1099. 10.1162/jocn_a_00393 23574539

[B26] Guterstam A. Petkova V. I. Ehrsson H. H. (2011). The illusion of owning a third arm. *PLoS One* 6:e17208. 10.1371/journal.pone.0017208 21383847 PMC3044173

[B27] Harvie D. S. Rio E. Smith R. T. Olthof N. Coppieters M. W. (2020). Virtual reality body image training for chronic low back pain: a single case report. *Front. Virtual Real.* 1:13. 10.3389/fnhum.2020.00013 32116602 PMC7033449

[B28] Hirakawa Y. Hara M. Fujiwara A. Hanada H. Morioka S. (2014). The relationship among psychological factors, neglect-like symptoms and postoperative pain after total knee arthroplasty. *Pain Res. Manag.* 19 251–256. 10.1155/2014/471529 25101335 PMC4197752

[B29] Howe L. C. Goyer J. P. Crum A. J. (2017). Harnessing the placebo effect: exploring the influence of physician characteristics on placebo response. *Health psychol.* 36 1074–1082. 10.1037/hea0000499 28277699 PMC7608626

[B30] Kalckert A. Perera A. T. Ganesan Y. Tan E. (2019). Rubber hands in space: the role of distance and relative position in the rubber hand illusion. *Exp. Brain Res.* 237 1821–1832. 10.1007/s00221-019-05539-6 31079236 PMC6584242

[B31] Kern A. Kramm C. Witt C. M. Barth J. (2020). The influence of personality traits on the placebo/nocebo response: a systematic review. *J. Psychosom. Res.* 128:109866. 10.1016/j.jpsychores.2019.109866 31760341

[B32] Kilteni K. Maselli A. Kording K. P. Slater M. (2015). Over my fake body: body ownership illusions for studying the multisensory basis of own-body perception. *Front. Hum. Neurosci.* 9:141. 10.3389/fnhum.2015.00141 25852524 PMC4371812

[B33] Lane T. Yeh S. L. Tseng P. Chang A. Y. (2017). Timing disownership experiences in the rubber hand illusion. *Cogn. Res. Princ. Implic* 2:4. 10.1186/s41235-016-0041-4 28203632 PMC5281674

[B34] Limanowski J. (2021). Precision control for a flexible body representation. *Neurosci. Biobehav. Rev.* 134:104401. 10.1016/j.neubiorev.2021.10.023 34736884

[B35] Louw A. Zimney K. Puentedura E. J. Diener I. (2016). The efficacy of pain neuroscience education on musculoskeletal pain: a systematic review of the literature. *Physiother. Theory Pract.* 32 332–355. 10.1080/09593985.2016.1194646 27351541

[B36] Ma K. Qu J. Yang L. Zhao W. Hommel B. (2021). Explicit and implicit measures of body ownership and agency: affected by the same manipulations and yet independent. *Exp. Brain Res.* 239 2159–2170. 10.1007/s00221-021-06125-5 33974114

[B37] Martini M. Kilteni K. Maselli A. Sanchez-Vives M. V. (2015). The body fades away: investigating the effects of transparency of an embodied virtual body on pain threshold and body ownership. *Sci. Rep.* 5:13948. 10.1038/srep13948 26415748 PMC4586459

[B38] Martini M. Perez-Marcos D. Sanchez-Vives M. V. (2013). What color is my arm? changes in skin color of an embodied virtual arm modulates pain threshold. *Front. Hum. Neurosci.* 7:438. 10.3389/fnhum.2013.00438 23914172 PMC3728482

[B39] Matamala-Gomez M. Diaz Gonzalez A. M. Slater M. Sanchez-Vives M. V. (2019). Decreasing pain ratings in chronic arm pain through changing a virtual body: different strategies for different pain types. *J. Pain* 20 685–697. 10.1016/j.jpain.2018.12.001 30562584

[B40] Matamala-Gomez M. Nierula B. Donegan T. Slater M. Sanchez-Vives M. V. (2020). Manipulating the perceived shape and color of a virtual limb can modulate pain responses. *J. Clin. Med.* 9:291. 10.3390/jcm9020291 31973014 PMC7074286

[B41] Meconi F. Doro M. Schiano Lomoriello A. Mastrella G. Sessa P. (2018). Neural measures of the role of affective prosody in empathy for pain. *Sci. Rep.* 8:291. 10.1038/s41598-017-18552-y 29321532 PMC5762917

[B42] Nierula B. Martini M. Matamala-Gomez M. Slater M. Sanchez-Vives M. V. (2017). Seeing an embodied virtual hand is analgesic contingent on colocation. *J. Pain* 18 645–655. 10.1016/j.jpain.2017.01.003 28108385

[B43] Nishigami T. Wand B. M. Newport R. Ratcliffe N. Themelis K. Moen D. (2019). Embodying the illusion of a strong, fit back in people with chronic low back pain. a pilot proof-of-concept study. *Musculoskelet. Sci. Pract.* 39 178–183. 10.1016/j.msksp.2018.07.002 30049618

[B44] Odermatt I. A. Buetler K. A. Wenk N. Özen Ö Penalver-Andres J. Nef T. (2021). Congruency of information rather than body ownership enhances motor performance in highly embodied virtual reality. *Front. Neurosci.* 15:678909. 10.3389/fnins.2021.678909 34295219 PMC8291288

[B45] Ogino Y. Nemoto H. Inui K. Saito S. Kakigi R. Goto F. (2007). Inner experience of pain: imagination of pain while viewing images showing painful events forms subjective pain representation in human brain. *Cereb. Cortex* 17 1139–1146. 10.1093/cercor/bhl023 16855007

[B46] Oldfield R. C. (1971). The assessment and analysis of handedness: the Edinburgh inventory. *Neuropsychologia* 9 97–113. 10.1016/0028-3932(71)90067-4 5146491

[B47] Olson J. A. Lifshitz M. Raz A. Veissière S. (2021). Super placebos: a feasibility study combining contextual factors to promote placebo effects. *Front. psychiatry* 12:644825. 10.3389/fpsyt.2021.644825 33746801 PMC7970115

[B48] Osumi M. Imai R. Ueta K. Nobusako S. Morioka S. (2014). Negative body image associated with changes in the visual body appearance increases pain perception. *PLoS One* 9:e107376. 10.1371/journal.pone.0107376 25210738 PMC4161431

[B49] Peerdeman K. J. van Laarhoven A. I. Donders A. R. Hopman M. T. Peters M. L. Evers A. W. (2015). Inducing expectations for health: effects of verbal suggestion and imagery on pain, itch, and fatigue as indicators of physical sensitivity. *PLoS One* 10:e0139563. 10.1371/journal.pone.0139563 26448183 PMC4598027

[B50] Perepelkina O. Vorobeva V. Melnikova O. Arina G. Nikolaeva V. (2018). Artificial hand illusions dynamics: onset and fading of static rubber and virtual moving hand illusions. *Conscious. Cogn.* 65 216–227. 10.1016/j.concog.2018.09.005 30218944

[B51] Pfister R. Klaffehn A. L. Kalckert A. Kunde W. Dignath D. (2021). How to lose a hand: sensory updating drives disembodiment. *Psychon. Bull. Rev.* 28 827–833. 10.3758/s13423-020-01854-0 33300113 PMC8219564

[B52] Riemer M. Bublatzky F. Trojan J. Alpers G. W. (2015). Defensive activation during the rubber hand illusion: ownership versus proprioceptive drift. *Biol. Psychol.* 109 86–92. 10.1016/j.biopsycho.2015.04.011 25960069

[B53] Romano J. Kromrey J. D. Coraggio J. Skowronek J. (2006). “Appropriate statistics for ordinal level data: should we really be using t-test and cohen’sd for evaluating group differences on the NSSE and other surveys?,” in *proceedings of the Annual Meeting of the Florida Association of Institutional Research*, Cocoa Beach, FL.

[B54] Rossettini G. Camerone E. M. Carlino E. Benedetti F. Testa M. (2020). Context matters: the psychoneurobiological determinants of placebo, nocebo and context-related effects in physiotherapy. *Arch. Physiother.* 10:11. 10.1186/s40945-020-00082-y 32537245 PMC7288522

[B55] Rossettini G. Carlino E. Testa M. (2018). Clinical relevance of contextual factors as triggers of placebo and nocebo effects in musculoskeletal pain. *BMC Musculoskelet. Disord.* 19:27. 10.1186/s12891-018-1943-8 29357856 PMC5778801

[B56] Sessa P. Meconi F. Han S. (2014). Double dissociation of neural responses supporting perceptual and cognitive components of social cognition: evidence from processing of others’ pain. *Sci. Rep.* 4:7424. 10.1038/srep07424 25502570 PMC4262888

[B57] Slater M. Perez-Marcos D. Ehrsson H. H. Sanchez-Vives M. V. (2008). Towards a digital body: the virtual arm illusion. *Front. Hum. Neurosci.* 2:6. 10.3389/neuro.09.006.2008 18958207 PMC2572198

[B58] Synofzik M. Vosgerau G. Newen A. (2008). I move, therefore I am: a new theoretical framework to investigate agency and ownership. *Conscious. Cogn.* 17 411–424. 10.1016/j.concog.2008.03.008 18411059

[B59] Ten Brink A. F. Halicka M. Vittersø A. D. Jones H. G. Stanton T. R. Bultitude J. H. (2021). Validation of the bath crps body perception disturbance scale. *J. Pain* 22 1371–1384. 10.1016/j.jpain.2021.04.007 33964412

[B60] Themelis K. Ratcliffe N. Nishigami T. Wand B. M. Newport R. Stanton T. R. (2021). The effect of visually manipulating back size and morphology on back perception, body ownership, and attitudes towards self-capacity during a lifting task. *Psychol. Res.* 10.1007/s00426-021-01609-z [Epub ahead of print]. 34727227 PMC9363286

[B61] Tinnermann A. Geuter S. Sprenger C. Finsterbusch J. Büchel C. (2017). Interactions between brain and spinal cord mediate value effects in nocebo hyperalgesia. *Science* 358 105–108. 10.1126/science.aan1221 28983051

[B62] Trojan J. Fuchs X. Speth S. L. Diers M. (2018). The rubber hand illusion induced by visual-thermal stimulation. *Sci. Rep.* 8:12417. 10.1038/s41598-018-29860-2 30127441 PMC6102275

[B63] Tsakiris M. (2008). Looking for myself: current multisensory input alters self-face recognition. *PLoS One* 3:e4040. 10.1371/journal.pone.0004040 19107208 PMC2603324

[B64] Tsakiris M. (2010). My body in the brain: a neurocognitive model of body-ownership. *Neuropsychologia* 48 703–712. 10.1016/j.neuropsychologia.2009.09.034 19819247

[B65] Tsakiris M. Carpenter L. James D. Fotopoulou A. (2010). Hands only illusion: multisensory integration elicits sense of ownership for body parts but not for non-corporeal objects. *Exp. Brain Res.* 204 343–352. 10.1007/s00221-009-2039-3 19820918

[B66] Tsakiris M. Haggard P. (2005). The rubber hand illusion revisited: visuotactile integration and self-attribution. *J. Exp. psychol. Hum. Percept. Perform.* 31 80–91. 10.1037/0096-1523.31.1.80 15709864

[B67] Uritani D. Ikeda A. Shironoki T. Matsubata K. Mutsura Y. Fujii T. (2021). Perceptions, beliefs, and needs of Japanese people with knee osteoarthritis during conservative care: a qualitative study. *BMC Musculoskelet. Disord.* 22:754. 10.1186/s12891-021-04641-7 34479525 PMC8417949

[B68] Wobbrock J. O. Findlater L. Gergle D. Higgins J. J. (2011). “The aligned rank transform for nonparametric factorial analyses using only anova procedures,” in *Proceedings of the SIGCHI Conference on Human Factors in Computing Systems (CHI ’11)*, New York, NY, 143–146. 10.1145/1978942.1978963

[B69] Wood L. Hendrick P. A. (2019). A systematic review and meta-analysis of pain neuroscience education for chronic low back pain: short-and long-term outcomes of pain and disability. *Eur. J. Pain* 23 234–249. 10.1002/ejp.1314 30178503

[B70] Zigmond A. S. Snaith R. P. (1983). The hospital anxiety and depression scale. *Acta Psychiatry. Scand.* 67 361–370. 10.1111/j.1600-0447.1983.tb09716.x 6880820

[B71] Zou K. Wong J. Abdullah N. Chen X. Smith T. Doherty M. (2016). Examination of overall treatment effect and the proportion attributable to contextual effect in osteoarthritis: meta-analysis of randomised controlled trials. *Ann. Rheum. Dis.* 75 1964–1970. 10.1136/annrheumdis-2015-208387 26882927 PMC5099197

