# Supplementary material for: Verbal Suggestion Modulates the Sense of Ownership and Heat Pain Threshold During the “Injured” Rubber Hand Illusion
Source: Front Hum Neurosci. 2022 Apr 25;16:837496. doi: 10.3389/fnhum.2022.837496 (PMC9082029; doi:10.3389/fnhum.2022.837496)
Supplement: Supplementary file 1 [file Table_1.DOCX]

Supplementary Material

TABLE S1. Basic Characteristics (Experiment 1 and Experiment 2)

|  | Experiment 1 | Experiment 2 | | |
| --- | --- | --- | --- | --- |
|  | Participant  (n＝15)  Mean (SD) | No-fear group  (n＝15)  Mean (SD) | Fear group  (n＝15)  Mean (SD) | Group  difference^＊^  p-value |
| Age (years) | 26.8 (5.5) | 26.0 (4.5) | 27.5 (4.4) | 0.16 |
| Gender (males/females) | 6/9 | 6/9 | 5/10 | 0.70 |
| Educational background (years) | 15.0 (0.7) | 15.5 (0.6) | 15.5 (0.6) | 1.00 |
| Handedness (right/left) | 12/3 | 14/1 | 14/1 | 1.00 |
| HADS Total score  　　 Anxiety  　　 Depression | 11.1 (4.5)  5.4 (3.0)  5.8 (2.6) | 11.1 (4.8)  5.5 (3.0)  5.7 (3.1) | 11.6 (5.2)  5.8 (3.3)  5.8 (3.1) | 0.80  0.72  0.80 |

^＊^In experiment 2, the group scores were compared with unpaired t-test (age), chi-square test (gender, handedness), and Mann-Whitney U test (educational background, HADS: total, anxiety, depression). Abbreviations: HADS, Hospital Anxiety and Depression Scale.
